# Supplementary material for: A systematic review of the limitations of large language models in generating healthcare content
Source: PLOS Digit Health. 2026 Apr 8;5(4):e0001354. doi: 10.1371/journal.pdig.0001354 (PMC13061218; doi:10.1371/journal.pdig.0001354)
Supplement: S1 Appendix — (DOCX) [file pdig.0001354.s001.docx]

| **S1 Appendix.** Quality assessment. | | | | | | | | | |
| --- | --- | --- | --- | --- | --- | --- | --- | --- | --- |
| **No.** | **Author(s)/ Citation** | **Q1** | **Q2** | **Q3** | **Q4** | **Q5** | **Q6** | **Score** |  |
|  | Barhom/[1] | 2 | 2 | 1 | 2 | 2 | 2 | 11 |  |
|  | Aliyeva/[2] | 1 | 1 | 1 | 1 | 2 | 2 | 8 |  |
|  | Alkuraya/[3] | 2 | 2 | 2 | 2 | 2 | 2 | 12 |  |
|  | Almutairi/[4] | 2 | 2 | 2 | 2 | 2 | 2 | 12 |  |
|  | Alshak/[5] | 2 | 2 | 2 | 2 | 2 | 2 | 12 |  |
|  | Asfuroğlu/[6] | 2 | 2 | 2 | 2 | 2 | 2 | 12 |  |
|  | Balas/[7] | 2 | 1 | 1 | 2 | 2 | 2 | 10 |  |
|  | Bazzari/[8] | 2 | 1 | 1 | 2 | 2 | 2 | 10 |  |
|  | Bentzen/[9] | 2 | 2 | 1 | 2 | 2 | 2 | 11 |  |
|  | Brewster/[10] | 2 | 2 | 1 | 1 | 2 | 2 | 10 |  |
|  | Buhr/[11] | 2 | 2 | 0 | 2 | 2 | 2 | 10 |  |
|  | Bull/[12] | 2 | 2 | 1 | 2 | 2 | 2 | 11 |  |
|  | Cankurtaran/[13] | 2 | 1 | 0 | 2 | 2 | 2 | 9 |  |
|  | Chen/[14] | 2 | 2 | 1 | 2 | 2 | 2 | 11 |  |
|  | Cuthbert/[15] | 2 | 2 | 1 | 2 | 2 | 2 | 11 |  |
|  | Darji/[16] | 2 | 2 | 1 | 2 | 2 | 2 | 11 |  |
|  | Daza/[17] | 2 | 2 | 1 | 2 | 2 | 2 | 11 |  |
|  | Dergaa/[18] | 2 | 2 | 0 | 2 | 2 | 0 | 8 |  |
|  | Eggmann/[19] | 2 | 1 | 1 | 2 | 2 | 2 | 10 |  |
|  | Elpasiony/[20] | 2 | 2 | 0 | 2 | 2 | 2 | 10 |  |
|  | Erkan/[21] | 2 | 2 | 0 | 1 | 2 | 1 | 8 |  |
|  | Fraile/[22] | 2 | 2 | 2 | 2 | 2 | 2 | 12 |  |
|  | Franc/[23] | 2 | 2 | 0 | 2 | 2 | 2 | 10 |  |
|  | Gaebe/[24] | 2 | 2 | 2 | 2 | 2 | 2 | 12 |  |
|  | Ge/[25] | 2 | 2 | 2 | 2 | 2 | 2 | 12 |  |
|  | Geneş/[26] | 2 | 2 | 1 | 2 | 2 | 2 | 11 |  |
|  | Gill/[27] | 2 | 2 | 0 | 1 | 2 | 1 | 8 |  |
|  | Grossman/[28] | 2 | 2 | 1 | 2 | 2 | 2 | 11 |  |
|  | Gupta/[29] | 1 | 1 | 1 | 2 | 2 | 2 | 9 |  |
|  | Gürbostan/[30] | 2 | 1 | 1 | 1 | 2 | 2 | 9 |  |
|  | Haider/[31] | 2 | 2 | 2 | 2 | 2 | 2 | 12 |  |
|  | Halaseh/[32] | 2 | 2 | 1 | 2 | 2 | 2 | 11 |  |
|  | Hristidis/[33] | 2 | 1 | 2 | 2 | 1 | 2 | 10 |  |
|  | Huang/[34] | 2 | 2 | 2 | 2 | 2 | 2 | 12 |  |
|  | Huang/[35] | 2 | 1 | 2 | 1 | 2 | 2 | 10 |  |
|  | Bartolucci/[36] | 2 | 2 | 1 | 1 | 2 | 2 | 10 |  |
|  | Jeyaraman/[37] | 1 | 1 | 0 | 2 | 1 | 2 | 7 |  |
|  | Jin/[38] | 2 | 2 | 2 | 2 | 1 | 2 | 11 |  |
|  | Joerg/[39] | 2 | 2 | 2 | 2 | 2 | 2 | 12 |  |
|  | Joshi/[40] | 1 | 2 | 2 | 2 | 2 | 2 | 11 |  |
|  | Kalam/[41] | 1 | 0 | 2 | 2 | 2 | 2 | 9 |  |
|  | Kaya Kaçar/[42] | 2 | 2 | 2 | 2 | 2 | 2 | 12 |  |
|  | Khosravi/[43] | 2 | 2 | 2 | 2 | 2 | 2 | 12 |  |
|  | Kıyak/[44] | 2 | 2 | 2 | 2 | 1 | 1 | 10 |  |
|  | Koh/[45] | 2 | 0 | 0 | 2 | 2 | 2 | 8 |  |
|  | Koh/[46] | 1 | 2 | 2 | 1 | 1 | 2 | 9 |  |
|  | Kong/[47] | 2 | 1 | 2 | 2 | 2 | 2 | 11 |  |
|  | Kral/[48] | 2 | 2 | 2 | 2 | 2 | 2 | 12 |  |
|  | Lang/[49] | 2 | 2 | 2 | 2 | 2 | 2 | 12 |  |
|  | Lavoie-Gagne/[50] | 2 | 2 | 2 | 2 | 2 | 1 | 11 |  |
|  | Lee/[51] | 1 | 2 | 1 | 1 | 2 | 1 | 8 |  |
|  | Lower/[52] | 1 | 2 | 2 | 1 | 1 | 2 | 9 |  |
|  | Maitland/[53] | 2 | 2 | 2 | 2 | 2 | 2 | 12 |  |
|  | Malik/[54] | 2 | 2 | 2 | 2 | 2 | 2 | 12 |  |
|  | Morath/[55] | 2 | 2 | 2 | 2 | 2 | 2 | 12 |  |
|  | Nguyen/[56] | 1 | 2 | 1 | 2 | 2 | 2 | 10 |  |
|  | Patel/[57] | 1 | 1 | 2 | 1 | 2 | 0 | 7 |  |
|  | Read/[58] | 2 | 1 | 2 | 1 | 2 | 2 | 10 |  |
|  | Riley/[59] | 2 | 2 | 2 | 2 | 2 | 2 | 12 |  |
|  | Rodgers/[60] | 2 | 2 | 1 | 2 | 2 | 2 | 11 |  |
|  | Şahin/[61] | 2 | 1 | 2 | 2 | 2 | 2 | 11 |  |
|  | Singh/[62] | 2 | 2 | 2 | 2 | 2 | 2 | 12 |  |
|  | Singh/[63] | 1 | 2 | 1 | 1 | 2 | 2 | 9 |  |
|  | Soroudi/[64] | 2 | 2 | 2 | 2 | 2 | 2 | 12 |  |
|  | Sovrano/[65] | 1 | 2 | 2 | 1 | 0 | 1 | 7 |  |
|  | Sparks/[66] | 1 | 0 | 2 | 2 | 2 | 0 | 7 |  |
|  | Stephan/[67] | 2 | 1 | 2 | 2 | 2 | 2 | 11 |  |
|  | Tam/[68] | 2 | 2 | 1 | 1 | 0 | 2 | 8 |  |
|  | Tarris/[69] | 2 | 2 | 2 | 1 | 2 | 2 | 11 |  |
|  | Temsah/[70] | 1 | 2 | 2 | 2 | 2 | 2 | 11 |  |
|  | Theophilou/[71] | 2 | 2 | 2 | 2 | 1 | 2 | 11 |  |
|  | Urbina/[72] | 2 | 2 | 2 | 2 | 2 | 2 | 12 |  |
|  | Vikan/[73] | 2 | 2 | 2 | 1 | 2 | 2 | 11 |  |
|  | Wang/[74] | 2 | 2 | 2 | 2 | 2 | 2 | 12 |  |
|  | Yang/[75] | 2 | 1 | 1 | 2 | 2 | 2 | 10 |  |
|  | Yaş/[76] | 2 | 2 | 2 | 2 | 2 | 2 | 12 |  |
|  | Yetkin/[77] | 2 | 2 | 2 | 2 | 2 | 2 | 12 |  |
|  | Yılmaz/[78] | 2 | 2 | 1 | 0 | 2 | 2 | 9 |  |
|  | Zaman/[79] | 0 | 1 | 2 | 2 | 0 | 2 | 7 |  |
|  | Zeller/[80] | 2 | 1 | 2 | 2 | 2 | 2 | 11 |  |
|  | Zheng/[81] | 2 | 2 | 2 | 2 | 2 | 2 | 12 |  |
| Scoring AACODS checklist questions: Yes =2, Can’t Tell= 1, No = 0 | | | | | | | | | |
| Q1: Authority: Is the author or source of the information reputable and trustworthy?  Q2: Accuracy: Is the information reliable, truthful, and correct?  Q3: Coverage: Does the information cover the topic comprehensively and sufficiently?  Q4: Objectivity: Is the information presented in an unbiased and balanced way?  Q5: Date: Is the information current and up-to-date?  Q6: Significance: Is the information relevant, important, and valuable to the topic? | | | | | | | | | |

**References:**

1. Ali, K., et al., *ChatGPT—A double‐edged sword for healthcare education? Implications for assessments of dental students.* European Journal of Dental Education, 2024. **28**(1): p. 206–211.

2. Aliyeva, A., E. Alaskarov, and E. Sari, *Postoperative management of tympanoplasty with ChatGPT-4.0.* The journal of international advanced otology, 2025. **21**(1): p. e241797.

3. Alkuraya, I.F. *Is artificial intelligence getting too much credit in medical genetics?* in *American Journal of Medical Genetics Part C: Seminars in Medical Genetics*. 2023. Wiley Online Library.

4. ALMutairi, M., et al. *Synthetic arabic medical dialogues using advanced multi-agent llm techniques*. in *Proceedings of The Second Arabic Natural Language Processing Conference*. 2024.

5. Alshak, M.N., et al., *Assessing ChatGPT Responses to Frequently Asked Patient Questions in Reconstructive Urology.* Urology Practice, 2025. **12**(4): p. 451–458.

6. Asfuroğlu, Z.M., H. Yağar, and E. Gümüşoğlu, *High accuracy but limited readability of large language model-generated responses to frequently asked questions about Kienböck’s disease.* BMC Musculoskeletal Disorders, 2024. **25**(1): p. 879.

7. Balas, M., D.T. Wong, and S.A. Arshinoff, *Artificial intelligence, adversarial attacks, and ocular warfare.* AJO International, 2024. **1**(3): p. 100062.

8. Bazzari, F.H. and A.H. Bazzari, *Utilizing ChatGPT in telepharmacy.* Cureus, 2024. **16**(1).

9. Bentzen, S.M., *Artificial Intelligence in Health Care: A Rallying Cry for Critical Clinical Research and Ethical Thinking.* Clinical Oncology, 2025. **41**: p. 103798.

10. Brewster, R.C., et al., *Performance of ChatGPT and Google Translate for pediatric discharge instruction translation.* Pediatrics, 2024. **154**(1): p. e2023065573.

11. Buhr, C.R., et al., *Assessing unknown potential—quality and limitations of different large language models in the field of otorhinolaryngology.* Acta Oto-Laryngologica, 2024. **144**(3): p. 237–242.

12. Bull, D. and D. Okaygoun, *Evaluating the Performance of ChatGPT in the Prescribing Safety Assessment: Implications for Artificial Intelligence-Assisted Prescribing.* Cureus, 2024. **16**(11).

13. Cankurtaran, R.E., et al., *Reliability and usefulness of ChatGPT for inflammatory bowel diseases: an analysis for patients and healthcare professionals.* Cureus, 2023. **15**(10).

14. Chen, J., et al., *Assessing the performance of ChatGPT in bioethics: a large language model’s moral compass in medicine.* Journal of medical ethics, 2024. **50**(2): p. 97–101.

15. Cuthbert, R. and A.I. Simpson, *Artificial intelligence in orthopaedics: can chat generative pre-trained transformer (ChatGPT) pass section 1 of the fellowship of the royal college of surgeons (trauma & orthopaedics) examination?* Postgraduate Medical Journal, 2023. **99**(1176): p. 1110–1114.

16. Darji, V.N., C.C. Liao, and D. Liao. *Automated Interpretation of Non-Destructive Evaluation Contour Maps Using Large Language Models for Bridge Condition Assessment*. in *2024 IEEE International Conference on Big Data (BigData)*. 2024. IEEE.

17. Daza, J., et al., *Evaluation of four chatbots in autoimmune liver disease: A comparative analysis.* Annals of Hepatology, 2025. **30**(1): p. 101537.

18. Dergaa, I., et al., *ChatGPT is not ready yet for use in providing mental health assessment and interventions.* Frontiers in Psychiatry, 2024. **14**: p. 1277756.

19. Eggmann, F. and M.B. Blatz, *ChatGPT: Chances and Challenges for Dentistry.* Compendium of Continuing Education in Dentistry (15488578), 2023. **44**(4).

20. Elpasiony, N.M.A., E.M. Sabek, and S.S.M. Ibrahim, *Chat generative pre-trained transformers era: pros and cons between nursing researchers in Egypt.* BMC nursing, 2025. **24**(1): p. 667.

21. Erkan, E.E., et al. *Understanding Large Language Model Performance in Question Answering: A Comparative Analysis of Semantic and Lexical Metrics*. in *International Conference on Intelligent and Fuzzy Systems*. 2025. Springer.

22. Fraile Navarro, D., et al., *Expert evaluation of large language models for clinical dialogue summarization.* Scientific reports, 2025. **15**(1): p. 1195.

23. Franc, J.M., et al., *Accuracy of a commercial large language model (ChatGPT) to perform disaster triage of simulated patients using the simple triage and rapid treatment (START) protocol: gage repeatability and reproducibility study.* Journal of Medical Internet Research, 2024. **26**: p. e55648.

24. Gaebe, K. and B. van der Woerd, *Evaluation of large language models as a diagnostic tool for medical learners and clinicians using advanced prompting techniques.* PLoS One, 2025. **20**(8): p. e0325803.

25. Ge, J., et al., *Development of a liver disease–specific large language model chat interface using retrieval-augmented generation.* Hepatology, 2024. **80**(5): p. 1158–1168.

26. Geneş, M., et al., *Artificial Intelligence in Cardiac Rehabilitation: Assessing ChatGPT's Knowledge and Clinical Scenario Responses.* Archives of the Turkish Society of Cardiology/Türk Kardiyoloji Derneği Arşivi, 2025(3).

27. Gill, B., et al., *ChatGPT is a promising tool to increase readability of orthopedic research consents.* Journal of Orthopaedics, Trauma and Rehabilitation, 2024. **31**(2): p. 148–152.

28. Grossman, S., T. Zerilli, and J.P. Nathan, *Appropriateness of ChatGPT as a resource for medication‐related questions.* British Journal of Clinical Pharmacology, 2024. **90**(10): p. 2691–2695.

29. Gupta, E. and V. Gupta. *A Comparative Analysis of ESP32 and ESP8266 for AI-Powered Applications*. in *2025 International Conference on Next Generation Communication & Information Processing (INCIP)*. 2025. IEEE.

30. Gürbostan Soysal, G., et al., *Evaluating the effectiveness of chatbots and traditional resources in patient education on dry eye disease.* Clinical and Experimental Optometry, 2025: p. 1–5.

31. Haider, S.A., et al., *Synthetic patient–physician conversations simulated by large language models: A multi-dimensional evaluation.* Sensors, 2025. **25**(14): p. 4305.

32. Halaseh, F.F., et al., *ChatGPT’s role in improving education among patients seeking emergency medical treatment.* Western Journal of Emergency Medicine, 2024. **25**(5): p. 845.

33. Hristidis, V., et al., *ChatGPT vs Google for queries related to dementia and other cognitive decline: comparison of results.* Journal of Medical Internet Research, 2023. **25**: p. e48966.

34. Huang, W., et al., *ChatGPT-Assisted Deep Learning Models for Influenza-Like Illness Prediction in Mainland China: Time Series Analysis.* Journal of Medical Internet Research, 2025. **27**: p. e74423.

35. Huang, Y., et al., *Integrative modeling enables ChatGPT to achieve average level of human counselors performance in mental health Q&A.* Information Processing & Management, 2025. **62**(5): p. 104152.

36. Incerti Parenti, S., et al. *Online patient education in obstructive sleep apnea: ChatGPT versus Google Search*. in *Healthcare*. 2024. MDPI.

37. Jeyaraman, M., et al., *ChatGPT in medical education and research: a boon or a bane?* Cureus, 2023. **15**(8).

38. Jin, Y., et al., *ChatGPT and mycosis–a new weapon in the knowledge battlefield.* BMC infectious diseases, 2023. **23**(1): p. 731.

39. Joerg, L., et al., *AI‐generated dermatologic images show deficient skin tone diversity and poor diagnostic accuracy: an experimental study.* Journal of the European Academy of Dermatology and Venereology, 2025.

40. Joshi, S., et al., *Ensuring accuracy and equity in vaccination information from ChatGPT and CDC: mixed-methods cross-language evaluation.* JMIR Formative Research, 2024. **8**: p. e60939.

41. Kalam, K.T., et al., *ChatGPT and mental health: Friends or foes?* Health Science Reports, 2024. **7**(2): p. e1912.

42. Kaya Kaçar, H., Ö.F. Kaçar, and A. Avery, *Diet quality and caloric accuracy in AI-Generated diet plans: A comparative study across chatbots.* Nutrients, 2025. **17**(2): p. 206.

43. Khosravi, T., Z.M. Al Sudani, and M. Oladnabi, *To what extent does ChatGPT understand genetics?* Innovations in Education and Teaching International, 2024. **61**(6): p. 1320–1329.

44. Kıyak, Y.S. and A.A. Kononowicz, *Case-based MCQ generator: a custom ChatGPT based on published prompts in the literature for automatic item generation.* Medical teacher, 2024. **46**(8): p. 1018–1020.

45. Koh, M.C.Y., et al., *The role of an artificial intelligence model in antiretroviral therapy counselling and advice for people living with HIV.* HIV medicine, 2024. **25**(4): p. 504–508.

46. Koh, S.J.Q., K.K. Yeo, and J.J.-L. Yap, *Leveraging ChatGPT to aid patient education on coronary angiogram.* Ann Acad Med Singap, 2023. **52**(7): p. 374–377.

47. Kong, M., et al., *Evaluation of the accuracy and safety of machine translation of patient-specific discharge instructions: a comparative analysis.* BMJ quality & safety, 2025.

48. Kral, J., et al., *Exploring the benefits and challenges of AI-driven large language models in gastroenterology: Think out of the box.* Biomedical Papers of the Medical Faculty of Palacky University in Olomouc, 2024. **168**(4).

49. Lang, S., et al., *Is the information provided by large language models valid in educating patients about adolescent idiopathic scoliosis? An evaluation of content, clarity, and empathy: The perspective of the European Spine Study Group.* Spine Deformity, 2025. **13**(2): p. 361–372.

50. Lavoie-Gagne, O.Z., et al., *Assessing the Usability of ChatGPT Responses Compared to Other Online Information in Hand Surgery.* HAND, 2025: p. 15589447251329584.

51. Lee, T.J., et al., *Evaluating ChatGPT responses on atrial fibrillation for patient education.* Cureus, 2024. **16**(6).

52. Lower, K., et al., *ChatGPT-4: transforming medical education and addressing clinical exposure challenges in the post-pandemic era.* Indian Journal of Orthopaedics, 2023. **57**(9): p. 1527–1544.

53. Maitland, A., R. Fowkes, and S. Maitland, *Can ChatGPT pass the MRCP (UK) written examinations? Analysis of performance and errors using a clinical decision-reasoning framework.* BMJ Open, 2024. **14**(3): p. e080558.

54. Malik, A., et al. *Confidential and protected disease classifier using fully homomorphic encryption*. in *2024 IEEE Conference on Artificial Intelligence (CAI)*. 2024. IEEE.

55. Morath, B., et al., *Performance and risks of ChatGPT used in drug information: an exploratory real-world analysis.* European Journal of Hospital Pharmacy, 2024. **31**(6): p. 491–497.

56. Nguyen, T., *ChatGPT in medical education: a precursor for automation bias?* 2024, JMIR Publications Toronto, Canada. p. e50174.

57. Patel, A. and A. Ajumobi, *Evaluating the Reliability of OpenAI’s ChatGPT-4 in Providing Pre-colonoscopy Patient Guidance.* Cureus, 2025. **17**(6).

58. Read, R. and M. Lukies, *The quality of information produced by ChatGPT about conditions managed by interventional radiologists.* Journal of Medical Imaging and Radiation Oncology, 2025.

59. Riley, G., et al., *Evaluating the fidelity of AI-generated information on long-acting reversible contraceptive methods.* The European Journal of Contraception & Reproductive Health Care, 2025. **30**(2): p. 74–77.

60. Rodgers, D.L., et al., *Artificial intelligence and the simulationists.* Simulation in Healthcare, 2023. **18**(6): p. 395–399.

61. Şahin, M.F., et al., *Evaluation of information accuracy and clarity: ChatGPT responses to the most frequently asked questions about premature ejaculation.* Sexual medicine, 2024. **12**(3): p. qfae036.

62. Singh, H., et al. *From Text to Treatment: Large Language Models in Clinical Practice and Medical Research*. in *2024 International Conference on Progressive Innovations in Intelligent Systems and Data Science (ICPIDS)*. 2024. IEEE.

63. Singh, S., et al., *Enhancing Patient Education on Cardiovascular Rehabilitation with Large Language Models.* Missouri Medicine, 2025. **122**(1): p. 67.

64. Soroudi, D., et al., *Comparing provider and ChatGPT responses to breast reconstruction patient questions in the electronic health record.* Annals of Plastic Surgery, 2024. **93**(5): p. 541–545.

65. Sovrano, F., K. Ashley, and A. Bacchelli. *Toward eliminating hallucinations: Gpt-based explanatory ai for intelligent textbooks and documentation*. in *CEUR Workshop Proceedings*. 2023. CEUR-WS.

66. Sparks, C.A., et al., *Inadequate performance of ChatGPT on orthopedic board-style written exams.* Cureus, 2024. **16**(6).

67. Stephan, D., et al., *AI in Dental Radiology—Improving the Efficiency of Reporting With ChatGPT: Comparative Study.* Journal of Medical Internet Research, 2024. **26**: p. e60684.

68. Tam, W., et al., *Nursing education in the age of artificial intelligence powered Chatbots (AI-Chatbots): are we ready yet?* Nurse Education Today, 2023. **129**: p. 105917.

69. Tarris, G. and L. Martin, *Performance assessment of ChatGPT 4, ChatGPT 3.5, Gemini Advanced Pro 1.5 and Bard 2.0 to problem solving in pathology in French language.* Digital Health, 2025. **11**: p. 20552076241310630.

70. Temsah, A., et al., *DeepSeek in healthcare: revealing opportunities and steering challenges of a new open-source artificial intelligence frontier.* Cureus, 2025. **17**(2).

71. Theophilou, E., et al. *Learning to prompt in the classroom to understand AI limits: A pilot study*. in *International conference of the Italian association for artificial intelligence*. 2023. Springer.

72. Urbina, J.T., P.D. Vu, and M.V. Nguyen, *Disability ethics and education in the age of artificial intelligence: identifying ability Bias in ChatGPT and gemini.* Archives of Physical Medicine and Rehabilitation, 2025. **106**(1): p. 14–19.

73. Vikan, M., et al., *Reflecting on LLM Support in Reflexive Thematic Analysis: An Exploratory Study.* Qualitative Health Research, 2025: p. 10497323251365211.

74. Wang, Y., Y. Chen, and J. Sheng, *Assessing ChatGPT as a medical consultation assistant for chronic hepatitis B: Cross-language study of English and Chinese.* JMIR Medical Informatics, 2024. **12**: p. e56426.

75. Yang, Z., et al., *Unveiling GPT-4V's hidden challenges behind high accuracy on USMLE questions: Observational Study.* Journal of Medical Internet Research, 2025. **27**: p. e65146.

76. Yaş, S., et al., *Assessing the role of large language models in adolescent idiopathic scoliosis care: a comparison between ChatGPT and Google Gemini.* Acta Orthopaedica et Traumatologica Turcica, 2025. **59**(4): p. 222.

77. Yetkin, N.A., et al. *Evaluating the Reliability and Quality of Sarcoidosis-Related Information Provided by AI Chatbots*. in *Healthcare*. 2025. MDPI.

78. Yılmaz, İ.b.E. and L. Doğan, *Talking technology: exploring chatbots as a tool for cataract patient education.* Clinical and Experimental Optometry, 2025. **108**(1): p. 56–64.

79. Zaman, M., *ChatGPT for healthcare sector: SWOT analysis.* International journal of research in industrial engineering, 2023. **12**(3): p. 221–233.

80. Zeller, N.P., et al., *Assessing Accuracy of Chat Generative Pre-Trained Transformer’s Responses to Common Patient Questions Regarding Congenital Upper Limb Differences.* Journal of Hand Surgery Global Online, 2025. **7**(4): p. 100764.

81. Zheng, Y., et al., *Enhancing diabetes self-management and education: a critical analysis of ChatGPT's role.* Annals of Biomedical Engineering, 2024. **52**(4): p. 741–744.
