# Supplementary material for: A systematic review of the limitations of large language models in generating healthcare content
Source: PLOS Digit Health. 2026 Apr 8;5(4):e0001354. doi: 10.1371/journal.pdig.0001354 (PMC13061218; doi:10.1371/journal.pdig.0001354)
Supplement: Attachment — Submitted filename: Authors` Response.docx [file pdig.0001354.s004.docx]

| **Comment** | **Response** |
| --- | --- |
| **Editor** |  |
| This is a timely systematic review that addresses an important and rapidly evolving topic in healthcare AI. The review is comprehensive and well motivated; however, several key issues should be addressed to strengthen its rigor and clarity. I suggest authors addressing reviewers' concerns accrodingly. Methodological transparency could be improved by providing reproducible, database-specific search strategies, clarifying quality and bias assessment methods, and importantly, justifying exclusion of conference literature. The discussion would benefit from deeper analytical integration. | We are thankful of the honorable editor for such a positive view on our paper. We have done our best to adhere to the reviewers` comments in order to improve our manuscript. Hope the honorable editor is satisfied with the revisions. Hopefully, our paper would provide valuable insights regarding the topic within the literature. With kind regards. |
| **Reviewer 1** |  |
| 1. This study only includes articles published in English. This is a significant limitation for a systematic review because valuable research in other languages, such as Chinese, German, and Arabic, is important in artificial intelligence and healthcare. Excluding these sources may skew our understanding of global issues, especially regarding translation quality. It is crucial to reassess this limitation or at least discuss its effects clearly. | As the honorable reviewer can acknowledge, the authors of the study selected the English language as source of the study research as the main language of the most journals in the prominent databases in the global stage is English. Moreover, due to the lack of understanding of other languages such as Chinese, German, … by the authors, selection of these languages could have led to bias and inaccurate data. However, as the honorable reviewer demanded, we have discussed such limitation in the limitation section of the manuscript (highlighted in yellow). |
| 2. The Discussion section primarily repeats the results without fully exploring the connections between the input, process, and output limitations. A more in-depth analysis is needed to understand how input limitations, like a lack of Arabic datasets, lead to specific output problems, such as variable translation quality. | As the honorable reviewer can acknowledge, an overall reporting the results in the discussion section is recommended by the PRISMA reporting guidelines for systematic reviews. In this regard, due to limitations of the volume of the manuscript, we focused on the most highly cited group of limitations (output limitations of LLMs) in the discussion section, discussing the limitations and proposing some strategies to address the limitations. However, the initial paragraph of the discussion section presents an overall analysis of the findings regarding input, process and output limitations of the LLMs. Regarding the lack of Arabic datasets, as the context of the study was medical settings, we have provided a sentence presenting flawed, biased or lack of datasets being the primary main cause of variabilities witnessed in the output quality of LLMs in healthcare settings, while multiple paragraphs present some strategies to enhance the output quality of LLMs(highlighted in yellow). Moreover, multiple other paragraphs are added to the discussion section presenting further insights regarding the limitations of LLMs and the existing solutions for them based on the existing evidence (highlighted in yellow). hope the honorable reviewer is satisfied with the revisions, as providing further discussions would have elongated the text of the manuscript and decreased its readability significantly. |
| 3. In the Conclusion and Limitations section (Section 4), it is important to state clearly that reliance on English publications is a major limitation. This focus could distort the findings and underreport language-specific biases. Future research should address this issue by including studies in other languages to improve the generalizability of the findings. | As reported earlier, a couple of sentences are presented in the limitations section of the manuscript addressing the comment issued by the honorable reviewer (highlighted in yellow). |
| 4. The authors should better emphasize the connection between input flaws and output problems. The text states that output issues "mainly resulted from flaws in input data," but it should elaborate on this point. For example, one could state, "Limited multimodal data processing (input flaw) leads to incomplete responses and neglected imaging descriptions (output limitation) in radiology contexts. "A Hybrid Autoencoder and Gated Recurrent Unit Model Optimized by Honey Badger Algorithm for Enhanced Cyber Threat Detection in IoT Networks”. This reference discusses a hybrid deep learning model in the context of IoT security and relates directly to the limitations in the review of large language models (LLMs).  5. It shows the need for specialized hybrid architectures and optimization algorithms, in contrast to the general-purpose LLMs discussed. This reference highlights a successful application in a specific field (cyber threat detection), presenting a counterexample to the weaknesses of general LLMs in complex fields such as healthcare.  6. The need to optimize parameters reflects the LLM issue regarding reliance on general training, which leads to gaps in accuracy and biases.This reference should be included in the Discussion section while analyzing the relationship between output limitations and flaws in model design. | We are thankful of the honorable reviewer for the suggested reference. In this regard, we have cited the reference in the last paragraph of the discussion section as suggested by the honorable reviewer (highlighted in yellow). furthermore, throughout the discussion section, we have presented the connection between the output limitations and input limitations, presenting that most of the output limitations are rooted in the input limitations. Hope the honorable reviewer is satisfied with the discussions as presenting further details elongates the manuscript and decreases its readability. |
| 7. The text notes that large language models (LLMs) are prone to generating plausible but incorrect or fabricated information, a phenomenon known as It is important to emphasize this term in bold for clarity. | Edited throughout the text as the honorable reviewer demanded (highlighted in yellow). |
| 8. In Section 6.5, it is helpful to reference the original work that defines the IPO model to maintain academic rigor. Although the model is introduced, clarifying its ties to system analysis will improve understanding. It is suggested that the authors check the alignments in the reference section and front issues and correct anything if needed. | The original work for the IPO model and its clarifications are added as the honorable reviewer demanded (highlighted in yellow). hope the honorable reviewer is satisfied with the revisions. With kind regards. |
| **Reviewer 2** |  |
| The search strategy is overly minimal (“Limitations” AND “Large language models” AND “Healthcare”), and Table 1 lists broad synonym groups without showing the actual Boolean-structured queries used in each database. For reproducibility, PRISMA requires database-specific queries (e.g., PubMed MeSH lines). | As the honorable reviewer can acknowledge, the terms issued by the honorable reviewer were the categories utilized within the search strategy of our research, not the actual terms which were presented in table 1. In this regard, these terms were selected as per the topic and main aim of our research which was to accumulate data regarding the limitations of LLMs in provision of healthcare content. Regarding the database-specific search queries, we tried our best to shorten the seemingly elongated text of the manuscript to enhance its readability. However, in order to adhere to the comment issued by the honorable reviewer, we have now added database-specific search queries as demanded by the honorable reviewer (highlighted in yellow). |
| There is no mention of PROSPERO registration. Given the systematic review methodology, the absence of protocol registration raises concerns about potential bias and post-hoc modifications. | As the honorable reviewer can acknowledge, registration of systematic reviews in PROSPERO is not mandatory for the authors despite its supposedly contribution in avoiding of duplicate researches, and adherence of the research to the original protocol; In this regard, we neglected such registration due to limitations in time and potential revision of the search strategy during the conduction of research. Therefore, we believed such registration was not necessary. However, if the honorable reviewer believes such registration is necessary, we are ready to register our review in case of such obligation. |
| The manuscript excludes conference papers and letters but does not justify these exclusions. Many influential AI/LLM limitations papers appear in conference proceedings (ACL, NeurIPS, AAAI). | Multiple sentences are added to the corresponding part of the manuscript addressing the comment issued by the honorable reviewer (highlighted in yellow). |
| While the IPO model is a general systems-analysis tool, its adoption here seems partially post-hoc; some limitations overlap categories (e.g., prompt issues can be both input and process). Consider clarifying the rationale for mapping each limitation to its category. | As the honorable reviewer can acknowledge, the labeling and categorization process of codes, sub-themes and themes during the thematic analysis was conducted by the perception, mindset and overall views of the study authors as detailed in the thematic analysis part of the methodology section of the manuscript. Therefore, the existence of overlaps in some of the items of the thematic analysis section is unavoidable and it depends on the overall personal view of each expert within the context. in this regard, as described extensively within the corresponding section of the manuscript, we categorized the items during the thematic analysis based on the overall perception and views of the study authors. However, in order to enhance clarity of the thematic analysis, as recommended by the honorable reviewer, we have provided multiple sentences in the corresponding part of the manuscript presenting the definition of each category presented in the thematic analysis (highlighted in yellow). Hope the honorable reviewer is satisfied with the revisions, as provision further data would have decreased readability and increased the volume of the already elongated manuscript. |
| The results extend to many pages with repetitive descriptions of similar limitations. While comprehensive, it compromises readability. A more synthesized narrative plus thematic tables would improve clarity. | As the honorable reviewer can acknowledge, the data presented in the thematic analysis should be reported in detailed as per the guidelines issued by PRISMA. In order to have a more synthesized data and to enhance readability, we have provided table 2 and figure 2. We believe providing further tables and figures would extensively increase the volume of the already elongated manuscript and decrease its readability significantly. Anyhow, we have already provided a figure (striking image) presenting the overall study findings in an abstract format as per the guidelines issued by the journal. We believe that the honorable reviewer has missed the uploaded striking image. Anyhow, we are ready to design further figures in case of any further demands by the honorable reviewer. |
| The figure 2 displays frequency counts but lacks: definition of how “number of references” was computed (e.g., per theme or per sub-theme), visual clarity (font size, grouping). | As the honorable reviewer can acknowledge, the items presented in the figure are sub-themes; hence, the number of reference counts are corresponding to the sub-themes. However, in order to enhance clarity, we have provided a sentence to the corresponding part of the manuscript further clarifying this issue of the readers (highlighted in yellow). Moreover, the font size and grouping are deemed to have sufficient visual quality and it needs to zoom in by the readers. In this regard, due to the large volume of the figure, the readers might mistakenly consider the image having a low quality, which is not true in reality. |
| Much of the discussion repeats results rather than integrating them with broader literature or offering conceptual insight. The section could more deeply analyze why certain limitations dominate and how emerging LLM architectures (e.g., multimodal models, RAG, domain-specific pretraining) may mitigate them. | As the honorable reviewer can acknowledge, due to the large amount of data presented by our study findings, in order to enhance readability of the paper, while presenting an overall analysis of our study findings with the findings presented by the previous literature, we decided to focus our data analysis on most prominent category of data reported by our study findings (output limitations). In this regard, while discussing and analyzing the data presented by our study findings with those findings in the previous literature within the context, we provided some solutions for each limitation. Above all, we have now added several paragraphs to the discussion section of the manuscript presenting further discussions and analysis of findings considering the data presented by the literature within the context (highlighted in yellow). hope the honorable reviewer is satisfied with the revisions, as providing further discussions would have elongated the text of the manuscript and decreased its readability significantly. |
| Recent advances such as GPT-5, Gemini 2.0, Qwen 2.5, smaller <13B models like Gemma and domain-specific models (Med-PaLM, MedGemma, LLaMA-family medical finetunes) are not discussed, though their capabilities meaningfully impact the identified limitations. | As the honorable reviewer can acknowledge, the main aim of our study was to present the limitations of LLMs presenting the newest data presented by the literature. Therefore, presenting data regarding the recent advancements of LLMs is deemed to be out of the study context and further increases the volume of the already elongated manuscript. However, in order to adhere to the comment issued by the honorable reviewer, we have now provided a new paragraph at the end of the discussion section citing the recent advances of LLMs, while presenting further rationale behind the usability of our study data for the beneficiaries despite the advances of newer versions of LLMs (highlighted in yellow). |
| The study acknowledges language restriction but omits several important limitations: no assessment of publication bias,heterogeneity across study types (chatbot assessments, narrative essays, technical papers), and limited granularity in separating limitations specific to clinical, educational, and administrative contexts | As the honorable reviewer can acknowledge, we have already presented the data regarding the level of bias within the included studies within the corresponding part of the manuscript (data quality, and abstract, highlighted in yellow). However, regarding heterogeneity and limited categorization of data specific to clinical, educational, …, we have now added a new sentence to the limitations section of the manuscript addressing the comment issued by the honorable reviewer (highlighted in yellow). |
| Numerous grammatical inconsistencies remain (e.g., “LLMS” instead of LLMs; “supposedly to be derived…”).\ | We have addressed the issues throughout the manuscript as the honorable reviewer demanded. Moreover, despite rewording the word “supposedly” (highlighted in yellow), usage of such wording was due to the adherence to the guideline of the journal demanding the authors to summarize the research in plain language. |
| The abstract has several duplicated or redundant phrases. | Edited as the honorable reviewer demanded. |
| Improve flow by reducing overly long sentences throughout the manuscript. | Edited throughout the text as the honorable reviewer demanded. |
| Ensure consistent referencing formatting (several citations appear as ranges with missing formatting). Citation [75] is listed as “!!! INVALID CITATION !!!” which must be corrected. 75. . !!! INVALID CITATION !!! [4, 5, 12, 24, 29, 30, 41, 43, 51, 57, 59, 62, 67-70]. | Edited and fixed as the honorable reviewer demanded. |
| Table 2 is overly long and may be better placed in an appendix. | As the honorable reviewer can acknowledge, as table two presents the overall findings of the analysis of study findings, presenting it as an appendix would decrease readability and clarity of the study findings significantly. Moreover, this table presents the outcomes of study data analysis in a short format which enhances readability of the paper. Therefore, we kindly demand the honorable reviewer to be satisfied with the current status of the table. |
| Some themes overlap significantly (e.g., “dependence on input quality” vs “dependence on prompt quality”). Consider merging or refining boundaries. | As discussed earlier, the outcome of thematic analysis and the seeming overlaps existed within the thematic analysis is unavoidable due to the comprehensiveness and complexity of the study data. In this regard, as discussed earlier, the current form of the thematic analysis was derived from an overall discussion among the study authors based on their overall understanding, perception and views regarding each theme, sub-theme and code. For example, regarding the input quality and the prompt quality, as the prompt is just a tiny part of the input given to the LLMs by the users, neglecting or merging it with a broader context of input quality as a whole seems to lead to negligence of the important issue of prompt quality. In this regard, we tried our best to present the data as precise and specific as possible in order to provide a precise analysis of the study findings referring to all of the important items of the study data. Moreover, omitting the important issue of prompt quality or merging it with the input quality seems to lead to some levels of bias in the reporting of data as it distorts the reporting of the important data regarding prompt quality. |
| Terminology such as “accuracy gap” should be defined more precisely. | While, a detailed explanation for accuracy gap is presented in the study findings in the corresponding part of the thematic analysis, a prominent example of accuracy gap is added to the phrase throughout the manuscript in order to enhance clarity as the honorable reviewer demanded (highlighted in yellow). |
| The authors mention using ChatGPT-4 to “rewrite the text,” which is appropriate, but PLOS typically requires specifying: which sections were rewritten, whether human authors verified accuracy. | We have addressed the issue presented by the honorable reviewer in the corresponding part of the manuscript (highlighted in yellow). hope the honorable reviewer is satisfied with the revisions. In this regard, due to the large volume of the manuscript, we couldn’t provide further data which deemed to lead to decrease in readability of the manuscript in a significant manner. With kind regards. |
| **Reviewer 3** |  |
| This is a great work. It is timely and important, and it has a lot of present and future implications on both healthcare practice and research. The coverage and classification of the LLM limitations and the awareness about them are essential for different backgrounds and academic levels of people involved in designing, implementing and end-using them. Going forward, this subject can not be avoided and no matter what the limitations and challenges are, we have to face them, deal with them and tame them. Thanks for sharing this important work and thanks for giving me the opportunity to review it. | We are thankful of the honorable reviewer for such a positive remark regarding our paper. Hope our paper provides valuable data for future readers in this context. |
| I learned a lot from reading it. The researchers have summarized many ideas from the literature that they have reviewed, that I would not have time to read it myself and add to my understanding. So I have to be extremely thankful. | We are again grateful of the honorable reviewer for such an inspiring remark on our paper. |
| One of the injustices that afflict this study is that it is tackling a huge subject. While reading it, at some points or sub-topics, I found myself thinking that every one limitation or challenge mentioned here is worth a separate study because of the depth of the ideas discussed. | We agree with the honorable reviewer regarding the comprehensiveness and broadness of the research topic. In this regard, since our paper was a systematic review, we tried our best to make our topic as broad as possible to be able to include and summarize as much as data existing withing the literature in a single sheet. As the honorable reviewer rightfully said, each of the data presented by the study has the potential to be presented in a separate study; in this regard, future researchers can utilize the findings of this study in order to conduct such studies providing data regarding each limitation in a detailed format. |
| I would like to put my comments in the following bullets, following quoting the manuscript text portions hoping that this will help in shaping the final publication in the best shape.  “Two independent evaluators screened the references and assessed quality of the selected studies using the Authority, Accuracy, Coverage, Objectivity, Date, and Significance (AACODS) checklist, which examines Authority, Accuracy, Coverage, Objectivity, Date, and Significance.” In this sentence, the evaluators assessed the Authority, Accuracy etc of what exactly? For readers with different backgrounds, the second subject of this sentence needs to be clearer. | We have now edited the sentence and omitted those redundant parts which had decreased readability of that part of the manuscript. In this regard, as the honorable reviewer can witness, the AACODS checklist was used by the evaluators in order to assess the quality of the included studies within the review which is a routine in systematic reviews and is based on PRISMA guideline for systematic reviews, as the honorable reviewer can acknowledge. |
| • “Boyatzis's qualitative thematic approach” again, for any reader, they would expect some elaboration on this approach anywhere in the manuscript. The approach has been mentioned four times, including the one in the references without at least a single line simple explanation about it. | Multiple sentences are added to the data analysis part of the methodology section of the manuscript describing the steps of conducting thematic analysis based on the Boyatzis`s approach (highlighted in yellow). |
| • “A total of 81 studies were included in the final analysis. The included studies were predominantly of high quality and demonstrated minimal risk of bias.” Looking for the methodology used to assess risk of bias. | As the honorable reviewer can witness, as detailed in the quality assessment part of the methodology and results section of the manuscript, the quality and risk of bias was assessed using the AACODS checklist. |
| • “The thematic analysis identified key themes: data limitations, dependence on input and prompt quality, accessibility issues, model design and architecture constraints, interaction challenges, response quality and comprehensiveness, and ethical, safety, and regulatory concerns.” Tables? I would suggest that multiple tables should replace the huge Table 2, to ensure that the text is shown in correspondence with the relevant table contents. | As the honorable reviewer can acknowledge, since the thematic analysis of data presented the entirety of the limitations of LLMs, we decided to present whole of the data in a single table in order to enhance clarity of the study findings for the readers. Presenting data corresponding to a single question, is deemed to be suited to be done via a single table or figure, in contrast with multiple ones. We believe each table/figure should present data to a particular research question. In this regard, as the thematic analysis of data is corresponding to a single broad question (limitations of LLMs), dividing the table into multiple short tables seem to decrease clarity of the study findings for the readers. Such comprehensive tables are usual in systematic reviews as the honorable reviewer can acknowledge. Therefore, we kindly demand the honorable reviewer to be content with the current status of the table. Anyhow, if it is deemed obligatory, we are ready to divide the table into multiple small tables. |
| • “The study identified multiple limitations of LLMs in healthcare, with output issues being most common.” Reading through the text and the table, I found it necessary to repeat the point of dividing Table 2 into more tables, and this might bring even more subtopics that deserve to be included in the tables.? | Addressed this comment in the previous answer to the comments. Regarding the sub-topics or seemingly sub-themes or themes, we believe that adding more subtopics would decrease clarity of the study data and the readability of the manuscript significantly for the readers. In this regard, conduction of thematic analysis is based on the overall discussion of the study authors regarding the content of the thematic analysis. In this regard, as the honorable reviewer can acknowledge, each expert can present its own version of thematic analysis based on his/her mindset and views. Therefore, we kindly demand the honorable reviewer to be content with the current status of thematic analysis. |
| • “However, these output problems mainly resulted from flaws in input data, emphasizing the crucial 60 role of input quality. The study also proposed strategies to address these challenges.” Table? | As the honorable reviewer can witness, we have presented the strategies during the discussion section. Presenting the data regarding the strategies via tables seem to elongate the already bulky manuscript and decrease its readability. However, since the strategies are presented for diverse forms of limitations, presenting all of the strategies is deemed impossible, since we believe that each table/figure should present data regarding a particular research question. |
| • “The search yielded 81 studies, and the quality of the included studies was presented to be predominantly high.” Quality assessment criteria? | As the honorable reviewer can acknowledge, as reported in the quality assessment part of the methodology section of the manuscript, the quality assessment was conducted using AACODS checklist. The utilized criteria in this regard is presented in the corresponding part of the manuscript in detail (highlighted in yellow). |
| • “The thematic analysis yielded a number of themes and sub-themes.” Tables? | As the honorable reviewer can witness, table 2 presents the results of the thematic analysis. |
| • “The results of the research found that while the main area of LLMs` limitations is corresponding to the outputs, and particularly the existing gaps in accuracy, these limitations are supposedly to be derived from the existing flaws in the input data.” Important conclusion to be emphasized in the discussion. | As the honorable reviewer can witness, the initial paragraph of the discussion section presents an analysis regarding this finding (highlighted in yellow). |
| • “The study also presented some strategies to overcome these limitations 80 based on the existing data within the literature.” Important future plan or recommendation. | As the honorable reviewer can witness, we have referred to this issue in the implications section of the manuscript in detail. |
| • “Large language models (LLMs) are sophisticated artificial intelligence (AI) systems developed through extensive training on vast corpora of text data, enabling them to generate outputs that closely resemble human language(1). These models have been widely utilized across diverse medical domains, including health informatics, medical imaging, clinical diagnostics, treatment planning, ophthalmology, oncology, and other specialized fields(2). This trend signifies their broad and growing integration into medical research and clinical practice. LLMs have become pivotal in healthcare by enhancing clinical decision support, diagnostics, medical education, and patient engagement(2-4). They improve diagnostic accuracy by analyzing extensive clinical data and medical literature, aiding in personalized treatment planning and patient care management(3).” Debatable, but understood as completely dependant on the reference. | We are thankful of the honorable reviewer. |
| “98 Notwithstanding the numerous advantages previously discussed, LLMs in healthcare exhibit 99 several critical limitations that must be addressed to ensure their safe and effective deployment. 100 In this regard, they are prone to generating plausible yet factually incorrect or fabricated 101 information, a phenomenon known as hallucination, which poses significant risks in clinical 102 settings(3, 6).” Table for limitations, hallucinations included. | As the honorable reviewer can witness, table 2 presents the data. |
| “Furthermore, LLMs often lack the depth of contextual understanding required to 103 accurately interpret complex medical scenarios, as they may fail to integrate multifaceted clinical 104 data or temporal information adequately(3).” Other limitations, lack of depth, lack of contextuality, failure to integrate clinical data, failure to integrate temporal information. Table? | As the honorable reviewer can witness, table 2 presents the entirety of the data regarding the limitations based on the findings from the previous studies. Moreover, as the honorable reviewer can acknowledge, number of tables and figures are limited since they would elongate the manuscript and decrease its readability. More importantly, the journal guidelines have limited the number of figures and tables. Therefore, providing figure or tables for each group of findings is deemed impossible. |
| “The development and application of LLMs are also 105 constrained by limited access to high-quality, diverse clinical datasets due to privacy, ethical, and 106 legal challenges(7). Ethical and legal issues such as bias, misinformation, data privacy, and 107 insufficient regulatory frameworks further challenge their adoption(8)” Constraints to LLM development and constraints to LLM adoption.  “Additionally, the opaque, 108 "black box" nature of these models undermines transparency and interpretability, complicating 109 trust and reliance by healthcare professionals(9).” Limitation table: opacity, black box, trust and reliance. | Addressed in the previous comment. |
| “Practical concerns include their substantial 110 computational and energy demands, which limit feasibility in resource-constrained 111 environments(3).” Practical limitations, table? | Addressed in the previous comment. |
| “Such limitations of LLMs pose significant challenges to their adoption and 112 utilization across various sectors of healthcare systems. In this regard, these challenges must be 113 critically addressed to enable the successful and comprehensive integration of LLM technologies 114 within healthcare infrastructure.” Consequences of limitations. Table or figure. | Addressed in the previous comment. |
| “115 While several review studies have aimed to delineate the limitations of LLMs in providing 116 healthcare content, there remains a significant gap in the literature regarding a comprehensive 117 and systematic presentation of these limitations for end-users” Practical limitations in LLMs, research gap in LLM limitations. | A paragraph in the introduction section presents the research gap and limitations of the LLMs based on the previous literature (highlighted in yellow). |
| “In this regard, a systematic 118 review categorized the limitations of LLMs into two primary domains: design and output.” In this sentence, “ a systematic review”, should not it be “this systematic review”? | As the honorable reviewer can acknowledge, this paragraph presents the data regarding the previous literature, not our study. Therefore, using ‘a systematic review’ is correct. |
| “Design 119 limitations included several items such as lack of optimization for the medical domain, data 120 transparency issues, and accessibility challenges.” Table? | Addressed in the previous comments regarding addition of tables/figures. |
| “Output limitations also included several items 121 such as non-reproducibility, incompleteness, inaccuracies, safety concerns, and biases(6).” Table? | Addressed in the previous comment. |
| “The 122 data generated from such research would be invaluable for technology developers to enhance 123 the quality of their models.” In the introduction, “such research”? Again, are we still referring to this research or recommending such research in our introduction? | The corresponding sentence is edited as the honorable reviewer demanded (highlighted in yellow). |
| “Additionally, healthcare policymakers and administrators could 124 leverage this information to make informed decisions about implementing these technologies 125 within their organizations, fully acknowledging their current constraints.” Definitely | We are thankful of the honorable reviewer. |
| “Moreover, future 126 researchers would benefit from this detailed framework by conducting focused investigations on 127 each identified limitation, thereby contributing further insights and advancing the field for 128 subsequent users and stakeholders.” I totally agree. | We are thankful of the honorable reviewer. |
| “” Why is the methods or material section put after the conclusion? Little mentioned about the methodology of the research under the ‘Results’. Figure-1 | As the honorable reviewer can acknowledge, this has been done due to the journal`s guidelines. The methodology section under the conclusion section presents the methodology of the research in a detailed format. |
| Figure 1 “Records removed before screening: Duplicate records removed (n =1798) Records marked as ineligible by automation tools (n =0) Records removed for other reasons (n =0)” Could you please elaborate on the automation tools? What are the other reasons? | As the honorable reviewer can acknowledge, such wording is based on the formal version of PRISMA diagram for systematic reviews, and since we had not used any automation tools, we presented the number as 0. There was also no other research to elaborate. |
| • “Figure 1. PRISMA diagram.” Would this be explained to give the lacking ‘Methods’ section? | As the honorable reviewer can witness, the methods section is provided under the conclusion section in detail. |
| • “In this regard, 20% of the included studies were published in 2023, 139 40% in 2024, and the remaining 40% in 2025.” “In this regard” this phrase was overused in this script. It could be replaced by different places in different places. What is the significance of the distribution of the studies onto the years? Can this be added to the research questions and given explanation for its significance? | We have edited the corresponding sentence of the manuscript as demanded by the honorable reviewer (highlighted in yellow). regarding the significance of the distribution, as such analysis is deemed to be out of the study context and is deemed to provide no value for the study readers, we kindly demand to not to add it to the research question. Furthermore, it elongates the manuscript further. |
| • “2.1. Data quality 181 The quality assessment of the included studies indicated that they were predominantly of high 182 quality, with an average score of 10. Approximately 34% of the studies achieved the highest 183 quality score of 12, while about 8% scored 7, reflecting lower quality relative to the other 184 included studies. Moreover, the level of bias in the included studies was generally low, with 185 approximately 59% of the studies exhibiting the minimal possible bias (Appendix 1. Quality 186 Assessment).” The assessments of both quality and bias need to be explained in the text, in addition to any table. Also, the table in Appendix one is very brief. It shows the questions for the tabulated criteria, but I believe it needs more elaboration. The evaluation is generally subjective in nature. | We have provided a new sentence in the corresponding part of the results section presenting further insights regarding level of bias in the included studies in a subjective manner (highlighted in yellow). |
| “There was also an identified over-reliance on imaging modalities such as 254 CT and MRI, without adequate customization for specific clinical contexts” This is very important point. I believe it deserves wider elaboration, but I understand that it might over expand the scope of this work. | We totally agree with the honorable reviewer that further elaboration of the issue expands the scope of the research and elongates the manuscript further, decreasing its readability significantly. |
| “255 Furthermore, LLMs like ChatGPT demonstrated an inability for critical thinking necessary to tailor 256 and guide patient management effectively” I believe that for an LLM to have critical thinking, they would make them more challenging than any challenge or limitation mentioned here. However, guiding their thinking by human supervised training, and training and more training would make them more useful. | We totally agree with the honorable reviewer. We have also presented similar strategies throughout the discussion section, as the honorable reviewer can acknowledge. |
| “Conversation tracking posed 265 further limitations: ChatGPT 3.5 lacked conversation memory due to privacy and browsing 266 restrictions, while ChatGPT 4.0, despite tracking conversations, inaccurately counted inquiries, 267 thereby undermining feedback and dialogue continuity” Conversation memory, and tracking accuracy would be one of the biggest future solutions for many of the challenges in LLM clinical use. | We totally agree with the honorable reviewer. |
| “. Accuracy 293 gaps were evident, with occasional imprecision, indecisiveness, hallucinations, irrelevant 294 information, and omissions of key clinical considerations, particularly for special populations 295 such as pregnant patients; fabricated references were also observed sporadically” I realize that fabricated references are a feature of inaccuracy, but I would still suggest that this might better be a separate sentence. | As the honorable reviewer can acknowledge, due to the comprehensiveness and complexity of the study data, we were enforced to categorize the limitations as broad as possible in order to enhance clarity and readability of the paper. Therefore, we kindly demand the honorable reviewer to be content with the current status of the manuscript regarding categorization of data. Anyhow, in order to adhere to the comment issued by the honorable reviewer, we have separated the sentences (highlighted in yellow). |
| “2.2.3.2. Ethical, safety, and regulatory concerns” This is one of the richest sections of the manuscript. I believe it deserves a separate table or tables. Focusing on keywords and their explanations. It would help a lot in this research to coin new definitions for many keywords to standardize the future study and tackling of the LLMs limitations and challenges | As the honorable reviewer can acknowledge, as discussed earlier in the comments extensively, provision of further details is deemed impossible due to the longevity of the text and high volume of the manuscript, and more importantly journal`s limitations on the number of tables/figures. |
| “Table 2. Thematic analysis of findings.” A repeated comment on this table is that it can have its text content converted to single keywords and this can even help create figure and charts from it. | As the honorable reviewer can acknowledge, due to the high volume of the manuscript, and more importantly absurdity of provision of such keywords within the results section of the research manuscripts, providing such keywords are deemed unnecessary. In this regard, future readers can read the results of the study and grasp the keywords out of the text themselves. |
| “In this regard, in line with our study findings, a recent review emphasized 347 that the taxonomy of limitations associated with large language models reveals a substantially 348 greater number of codes related to output issues compared to those connected with design or 349 input phases.” Again, “in this regard”, I would suggest that the researchers could take the opportunity to claim the taxonomy of LLM limitations and standardize their linguistic use. | We have edited the corresponding text of the manuscript as the honorable reviewer demanded (highlighted in yellow). |
| “Figure 2. Distributions of limitations of LLMs, categorized according to the frequency of citations reported in the literature.” Again, I would recommend dividing this figure into three figures. It looks very crowded, and the text font is very tiny because of that. | As the honorable reviewer can acknowledge, and as discussed earlier, since the data presented in the figure is corresponding to a single research question (limitations of LLMs), presenting the data through multiple figures would make the manuscript vague and lower its understandability for the readers. More importantly, as the honorable reviewer can acknowledge, in the published version of the manuscript, readers can open the figure in a new tab in their browsers to have the figure in a high quality resolution. Therefore, we kindly demand the honorable reviewer to be content with the current status of tables and figures, as the journal limits the number of tables and figures also. |
| “381 8). Moreover, flawed, biased, or incomplete data can propagate inaccuracies and inconsistencies 382 within the model outputs, reducing their reliability and safety, especially in healthcare 383 applications” To explain this again. I give the researchers the richness of their work, but the bias and quality standards need to be explained so the reader can benefit from the manuscript and build upon it in improving their future clinical interactions with the LLMs | While, thanking the honorable reviewer for such a positive comment, as discussed earlier, provision of further data elongates the already bulky manuscript and decreases the readability of the manuscript significantly. Since the main aim of the study was to provide a comprehensive list of limitations of LLMs in provision of healthcare content, we did our best to focus the data provided by our research on the limitations and some strategies to overcome the limitations. Provision of detailed data regarding other aspects of LLMs seem to be out of the study context. The volume of data provided by a single research is limited and future studies need to be conducted to further elaborate data on the subject, as the honorable reviewer can acknowledge. Therefore, we kindly demand the honorable reviewer to be content with the current status of the manuscript, since we have provided further elaborations on the study data in the latest round of revisions. Provision of further data elongates the manuscript and decreases its readability significantly. |
| “. This finding also has significant 445 implicationsfor the beneficiaries. In this regard, our study proposed several strategies to address 446 major issues encountered by the LLMs in the provision of healthcare content.” Thanks again, | We are thankful of the honorable reviewer for such precise and detailed comments regarding our paper. Hope the honorable reviewer is satisfied with the revisions, as we have provided further data within our paper in the current round of revision. Providing further data seems to elongate the text of the manuscript significantly and decreases its readability. Hope our paper provides valuable data within the literature. With kind regards. |
